# Classification of cannabis strains in the Canadian market with discriminant analysis of principal components using genome-wide single nucleotide polymorphisms

**DOI:** 10.1371/journal.pone.0253387

**Published:** 2021-06-28

**Authors:** Dan Jin, Philippe Henry, Jacqueline Shan, Jie Chen

**Affiliations:** 1 Department of Biomedical Engineering, University of Alberta, Edmonton, Alberta, Canada; 2 PBG BioPharma Inc., Leduc, Alberta, Canada; 3 Egret Bioscience Ltd., West Kelowna, British Columbia, Canada; 4 Lighthouse Genomics Inc., Salt Spring Island, British Columbia, Canada; 5 Department of Electrical and Computer Engineering, University of Alberta, Edmonton, Alberta, Canada; National Cheng Kung University, TAIWAN

## Abstract

The cannabis community typically uses the terms “Sativa” and “Indica” to characterize drug strains with high tetrahydrocannabinol (THC) levels. Due to large scale, extensive, and unrecorded hybridization in the past 40 years, this vernacular naming convention has become unreliable and inadequate for identifying or selecting strains for clinical research and medicinal production. Additionally, cannabidiol (CBD) dominant strains and balanced strains (or intermediate strains, which have intermediate levels of THC and CBD), are not included in the current classification studies despite the increasing research interest in the therapeutic potential of CBD. This paper is the first in a series of studies proposing that a new classification system be established based on genome-wide variation and supplemented by data on secondary metabolites and morphological characteristics. This study performed a whole-genome sequencing of 23 cannabis strains marketed in Canada, aligned sequences to a reference genome, and, after filtering for minor allele frequency of 10%, identified 137,858 single nucleotide polymorphisms (SNPs). Discriminant analysis of principal components (DAPC) was applied to these SNPs and further identified 344 structural SNPs, which classified individual strains into five chemotype-aligned groups: one CBD dominant, one balanced, and three THC dominant clusters. These structural SNPs were all multiallelic and were predominantly tri-allelic (339/344). The largest portion of these SNPs (37%) occurred on the same chromosome containing genes for CBD acid synthases (CBDAS) and THC acid synthases (THCAS). The remainder (63%) were located on the other nine chromosomes. These results showed that the genetic differences between modern cannabis strains were at a whole-genome level and not limited to THC or CBD production. These SNPs contained enough genetic variation for classifying individual strains into corresponding chemotypes. In an effort to elucidate the confused genetic backgrounds of commercially available cannabis strains, this classification attempt investigated the utility of DAPC for classifying modern cannabis strains and for identifying structural SNPs.

## Introduction

Cannabis has a complex breeding history. Whether its botanical classification is monotypic (*sativa*) or polytypic (*sativa* and *indica*) remains controversial [1]. Since the 1980s, breeding for high psychoactive THC content has occurred very aggressively in North America [2]. Nearly all drug-type cannabis currently cultivated in the USA, Canada, and Europe are hybridized, resulting in thousands of strains [3]. Recent genetic studies focused on validating the vernacular classification of “Sativa” and “Indica” [4–7]. However, this terminology is inadequate for identifying or selecting strains for clinical research and medicinal production due to the misuse of the botanical nomenclature, extensive cross-breeding, and unreliable labelling during unrecorded hybridization [2]. One genetic study found that the reported ancestry percentage of “Sativa” vs. “Indica” for 81 drug stains is only moderately correlated with the calculated genetic structure (r^2^ = 0.36) [5]. In addition, CBD dominant strains and balanced strains (THC ≈ CBD), which have gained increasing attention due to CBD’s use as a therapeutic [8–12], have been omitted in recent classification studies.

Cannabis has a diploid genome (2n = 20) with nine autosomal chromosomes and one pair of sex chromosomes [13]. The length of the haploid genome size is 818 Mbp for females and 843 Mbp for males [14]. An SNP is a variation of a single nucleotide at a specific position in the genome, and it is useful for understanding the genetic basis of diversity among populations [15]. SNPs are usually bi-allelic, with two alleles observed in the population [16]. Multiallelic SNPs have more than one alternative allele for that locus. Tri-allelic SNPs, which have three nucleotide substitution-based alleles at the same position, are relatively rare but are being considered of great relevance in epidemiological studies [17], in disaster victim identification using mixed and/or degraded DNA samples [18], and in animals pedigree accuracy studies [19]. Tri-allelic SNPs are reported to have a higher power of discrimination than bi-allelic SNPs requiring fewer markers and lowering costs [18, 20]. However, tri-allelic SNPs have been excluded in cannabis population structural analysis in the current literature [6, 21].

Cannabis classification studies that employ SNPs generally used partial genome information with few or no overlap sequences between datasets [22]. Whole-genome sequencing is used less often in the literature, but is preferable despite its higher cost because it enables comparison of genome datasets from different sources [22]. It also provides comprehensive genetic information [22], as studies showed that differences between fiber- and drug-type cannabis are at a genome-wide level and not necessarily limited to genes involved in THC production [5]. The recent release of the 10-chromosome map of the cannabis genome [23–27] may improve the understanding of the genetic architecture, identify a superior set of SNPs associated with interesting traits, and reduce future targeted genotyping costs by using fewer but more accurate SNPs [28].

Several approaches are now available for the analysis of population genetic structure. One of these approaches is the DAPC, which is a multivariate clustering method that combines the merits of both principal component analysis (PCA) and discriminant analysis (DA) [7, 29–31]. PCA is a multivariate analysis that can be applied to large datasets to reduce dimensions, but does not provide a group assessment, which is essential for investigating genetic structures of biological populations [32]. DA achieves the best classification of individuals into pre-defined groups by maximizing between-group variation and minimizing within-group variation, but the number of variables (alleles) needs to be fewer than the number of observations (individuals), which is generally not the case for SNP data [29]. DAPC first uses PCA to transform raw data (genome-wide identified SNPs) into principal components (PC), which are mutually orthogonal linear combinations of the original variables. This ensures that variables submitted to DA are perfectly uncorrelated and that there are fewer variables than number of individuals. Then, linear discriminant functions, which are synthetic variables of linear combinations of these SNPs, are constructed to maximize inter-cluster differences and minimize intra-cluster variation [29]. By combining the advantages of PCA and DA, DAPC can identify groups, assign individuals to groups, visualize between-population differentiation, and identify individual alleles that have contributed to population structuring.

The objectives of this study are to:

1. investigate whether modern cannabis strains can be classified and differentiated at the whole-genome level, and
2. investigate the chromosomal location and putative functions of identified structural SNPs.

This study is a part of an integrated cannabis strain classification project utilizing genetic, chemical, and morphological profiles, wherein plants were grown in a commercial greenhouse under the same condition.

## Materials and methods

### DNA extraction and whole genome sequencing

This study included 23 commercially available cannabis strains, and the research was carried out under a cannabis research license issued by Health Canada. Where possible, the reported ancestry (“Sativa”, “Indica”, or “Sativa-dominant” and “Indica-dominant”) was obtained from the licensed producer providing the strain or from an online strain database (https://www.leafly.ca) (Table 1). Each strain was analyzed for chemical composition using methods established in our previous study [33] and labelled as “THC dominant”, “balanced”, and “CBD dominant”. DNA was extracted from 100 mg of fresh leaves for each strain using a Qiagen DNeasy Plant Mini Kit (QIAGEN, Canada). DNA concentrations were determined using a Qubit Fluorometer (Thermo Fisher Scientific, US). DNA integrity was tested by agarose gel electrophoresis. Library construction and sequencing were performed by BGI (USA) using DNBseq^™^ sequencing technology to a depth of 30x. DNBseq^™^ is a high-throughput sequencing solution, where DNA is fragmented into 100–300 bp and made into DNA nanoballs (DNB^™^), which are continuous DNA molecule with multiple head-to-tail copies of the same DNA fragment by linear isothermal rolling-circle replication. They are loaded onto high-density sequencing templates and sequenced by combinatorial probe-anchor synthesis (cPAS), where fluorescently tagged nucleotides complete for addition to the growing chain. After the addition of each nucleotide, high-resolution digital imaging is carried out where the DNB clusters are excited by a light source and a characteristic fluorescent signal is emitted. Hundreds of and thousands of clusters are sequenced in a massively parallel process. The emission wavelength, along with the signal intensity, determines the base call and the number of the cycles determines the length of the read. Sequence reads were then aligned to the reference genome assembly ASM23057v4 of a drug type strain Purple Kush (PK) in the NCBI BioProject database under accession number PRJNA73819 [34] using Burrows-Wheeler Alignment (BWA) tool [35]. New assignments of chromosomes numbers (1–10) were used as in ASM23057v5 [36]. The first step of SNP calling is marking duplications in BAM format files, and selected duplications are included in SNP calling by GATK (Genome Analysis Toolkit) (https://www.broadinstitute.org/gatk/). Local realignment around inDels is performed to avoid the bias of SNP calling, and the variation sites around inDel are identified as SNPs. A total of 235,334 SNPs was identified, including 225,046 bi-allelic and 10,288 multiallelic SNPs. After filtering for SNPs with no missingness by locus and a minor allele frequency less than 10% using VCFtools, 137,858 SNPs, including 128,810 bi-allelic and 9,048 multiallelic SNPs, remained for analysis.

**Table 1:** Strain information of 23 strains and preassigned clusters by DAPC.

Strain number	Strain name	Chemotypes	Clusters (W-SNPs)	Clusters (I-SNPs)	"Sativa" or "Indica"
**1**	Lemon Garlic OG	1-Balanced	C1	C4	"Indica" dominant
**2**	Royal Medic	2-Balanced	C3	C2	"Sativa" dominant
**3**	Blue Hawaiian	3-CBD	C3	C1	"Sativa" dominant
**4**	Kandy Kush	4-CBD	C3	C1	"Sativa" dominant
**5**	Special	5-CBD	C3	C1	Not provided
**6**	NN	6-CBD	C3	C1	Not provided
**7**	Dance World	7-Balanced	C3	C2	"Sativa" dominant
**8**	Treat	8-CBD	C3	C1	Not provided
**9**	High	9-Balanced	C3	C2	Not provided
**10**	CB7	10-CBD	C3	C1	Not provided
**11**	33°	11-THC	C1	C4	Not provided
**12**	Banana Cake	12-THC	C2	C5	"Indica" dominant
**13**	Bananium	13-THC	C3	C3	"Indica" dominant
**14**	Burmese Blueberry	14-THC	C2	C5	"Indica" dominant
**15**	Divine Banana	15-THC	C2	C4	"Indica" dominant
**16**	Granddaddy Purple	16-THC	C2	C5	"Indica" dominant
**17**	Lemon Love	17-THC	C1	C5	"Indica" dominant
**18**	Lemon Sorbet	18-THC	C1	C4	"Indica" dominant
**19**	MeatHead	19-THC	C2	C5	"Indica" dominant
**20**	Nanitro	20-THC	C1	C4	"Indica" dominant
**21**	Platinum Jelly Punch	21-THC	C1	C4	"Indica" dominant
**22**	SBSK2 (Lemon Thai)	22-THC	C3	C3	50/50 hybrid
**23**	Super sherbet	23-THC	C1	C4	"Indica" dominant

*The column of clusters W-SNPs was obtained using the whole set of 137,858 filtered SNPs. The column of clusters I-SNPs was obtained using 344 structural SNPs.

### Analysis of population structure and identification of structural SNPs

The population structure in this work was analyzed by DAPC using the *adegenet* package [37] in R software [38]. First, the *find*.*clusters* function ran successive K-means [39] for a range of *k* values (where the number of clusters *k* = K), and identified the optimal number of clusters by comparing the Bayesian Information Criterion (BIC) [40] of the corresponding models. After groups were assigned, a cross-validation function (*xvalDapc*) was used to determine the optimal number of PCs to avoid over-sacrificing information or over-fitting in the subsequent DAPC. In cross-validation, the data were divided into a training set (90% of the data) and a validation set (10% of the data) by default. DAPC was carried out on the training set and the accuracy of predicting the membership of individuals in the validation set was used to identify the number of PCs. The sampling and DAPC were repeated 30 times by default at each level of PC retention. After assigning individuals to clusters, DA was carried out on the retained PCs and contributions of the alleles to each discriminant function were stored. An SNPZIP analysis (*snpzip*) in R was then used to provide objective delineation between structural and non-structural SNPs, as identified by DAPC, to determine which SNPs contribute significantly to the between-population structure [41].

First, the whole set of 137,858 SNPs were applied to DAPC to identify SNPs that contributed most to the identified clusters. DAPC was carried out again using the identified SNPs to validate their differentiation efficiency by confirming the separation of the 23 strains into their preassigned clusters. A short sequence (about 600 nt) around each one of these identified SNP was searched using the BLAST software (https://blast.ncbi.nlm.nih.gov) against *Cannabis sativa* Annotation Release 100 [42]. In addition to DAPC, other clustering methods, including PCA, neighbor-joining (NJ) tree [43], and hierarchical dendrogram using Ward’s minimum variance method [44], were also employed to assess the robustness of the final inferred clusters. PCA and NJ tree were plotted using R. The hierarchical dendrogram was plotted using JMP 14.0.0.

## Results and discussions

### Discriminant analysis of principal components using 137,858 SNPs

**Fig 1:**
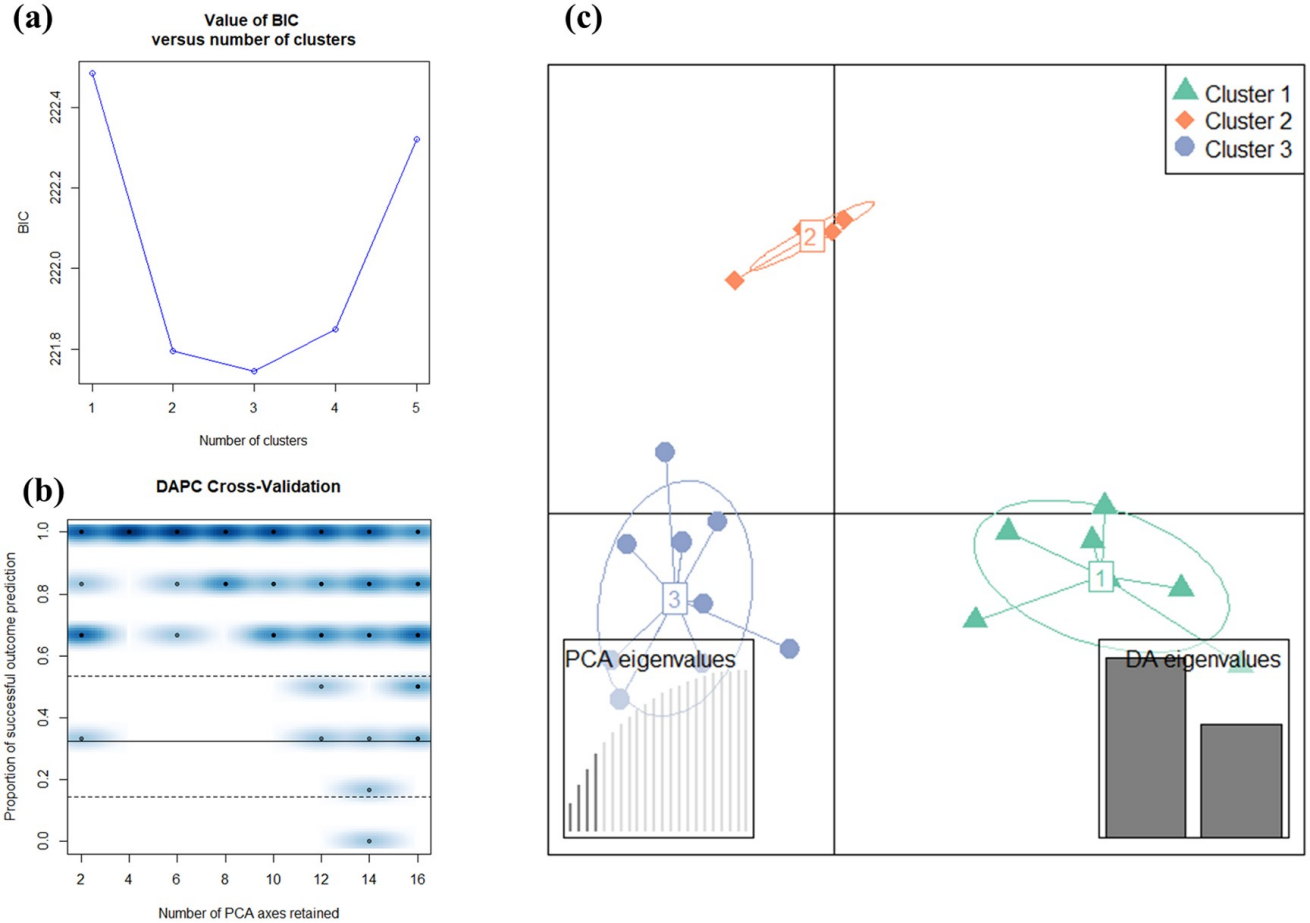
DAPC for 23 cannabis genotypes. (a) The x-axis is the number of clusters *k* and the y-axis is the corresponding value of BIC. (b) The plot of DAPC cross-validation. The x-axis is the number of PCA axes retained for DAPC, and the y-axis is the proportion of successful outcome prediction. Individual replicates appear as points, and the density of those points in different regions of the plot is displayed in blue. (c) DAPC plot for 23 cannabis genotypes along two linear discriminants (LD 1 and LD 2).

As indicated by the elbow in the curve of BIC values as a function of *k* in Fig 1(a), the optimal number of identified clusters was three, corresponding to the lowest BIC values. The number of PCs retained for DAPC analysis was four, as calculated by cross-validation in Fig 1(b), where it had 100% predictive success, and 0% associated root mean squared error (RMSE). In this study, the number of PCs associated with the highest mean success was also associated with the lowest MSE, which made it easier to choose the number of PCs to retain. For the subsequent DAPC analysis, four PCs and two discriminant functions were retained. The DAPC plot of 23 cannabis genotypes is shown in Fig 1(c). The grouping assignment for individual strains by DAPC is listed in Table 1 (as W-SNPs). C1 is a THC dominant cluster and includes six THC dominant strains (11, 17, 18, 20, 21, and 23-THC) and one balanced strain (1-balanced). C2 is another THC dominant cluster and includes five THC dominant strains (12, 14, 15, 16, and 19-THC). C3 is a cluster dominated by CBD dominant and the balanced strains which includes six CBD dominant strains (3, 4, 5, 6, 8, and 10-CBD), three balanced strains (2, 7, and 9-balanced), and two THC dominant strains (13 and 22-THC). While C2 is closer to C3 and is more distant to C1, C1and C3 are clearly separated along linear discriminant 1 (LD1). While C1 and C3 are roughly at the same level with respect to linear discriminant 2 (LD2), C2 is separated from both. PCA was also carried out on the same set of SNPs and results are shown in S1 Fig. Twenty-three cannabis strains are plotted along pair-wise PCs of the first 4 PCs, which account for 18.4%, 11.5%, 9.5%, and 8.7% of the total variance, respectively. Similarly, the first PC suggests the existence of a relatively compact CBD & balanced clade on the left side of the plot and a more dispersed THC dominant clade on the right side of the plot. Balanced strains share a closer gene pool with CBD dominant strains, while the THC gene pool is more dispersed. Because THC is psychoactive and its potency can be readily assessed through consumption, selection for increasing THC content started early and widely for recreational purposes by traditional breeding [45]. In contrast, CBD is non-psychoactive and must be analyzed in a laboratory for potency, and therefore breeding for high CBD concentrations began later [45]. A complete genome assembly implied that CBD dominant varieties were generated by integrating hemp-type CBD acid synthase gene clusters into a background of drug-type cannabis to elevate CBDA production [24]. These balanced strains may have been created by crossing purebred THC dominant types with CBD dominant types [46]. Therefore, there may be a relatively limited selection of CBD dominant strains for breeding balanced strains.

### Discriminant analysis of principal components using 344 structural SNPs

**Fig 2:**
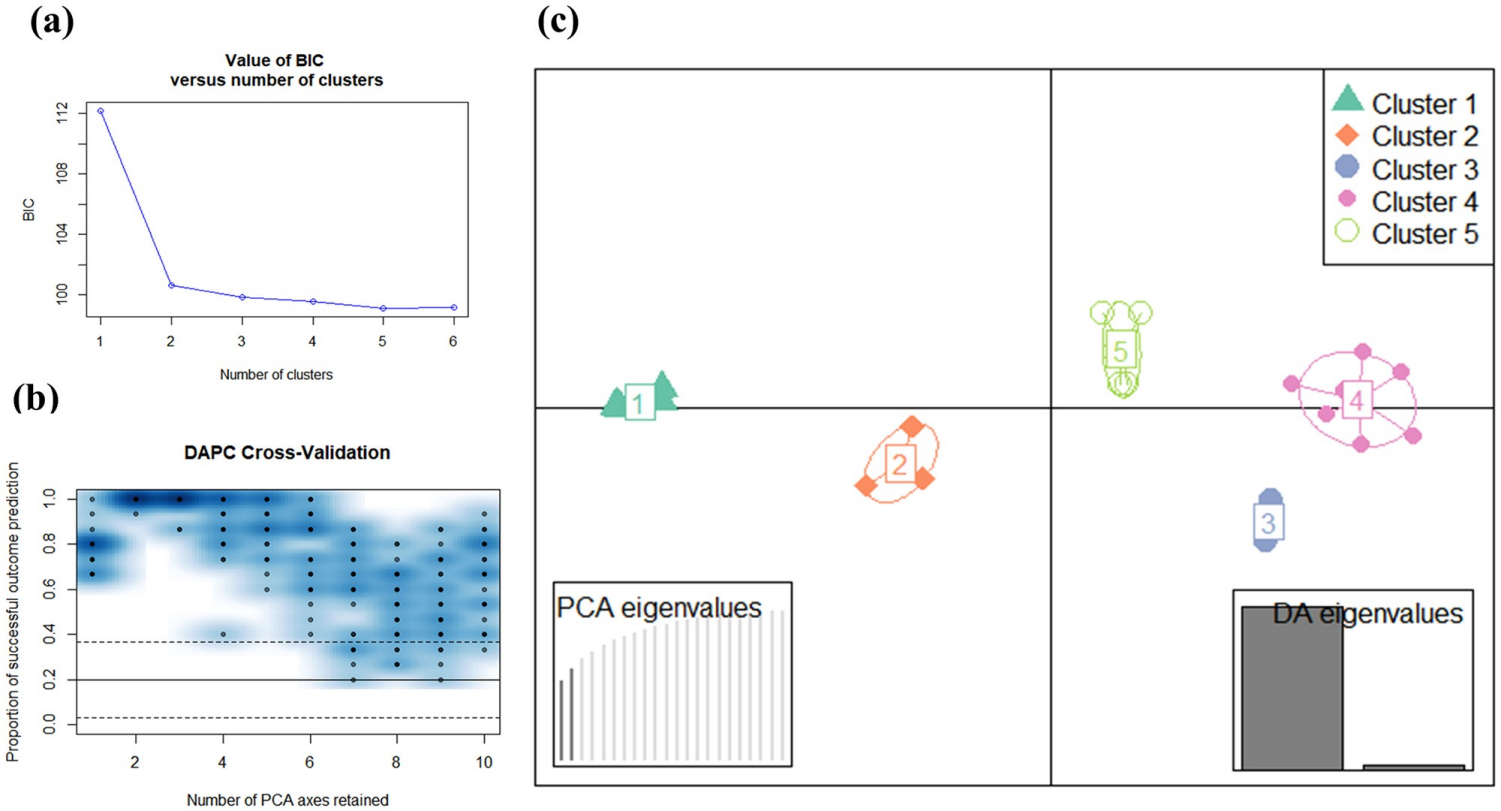
DAPC of 23 cannabis genotypes using 344 multiallelic structural SNPs. Clusters indicated as C1, C2, C3, C4, and C5 corresponds to the I-SNPs in Table 1.

DAPC was repeated using identified 344 structural SNPs. The optimal number of identified clusters was five, corresponding to the lowest BIC values (Fig 2(a)). Two PCs were retained for the following DAPC analysis in Fig 2(b), where it had 98.9% predictive success and 0.04% RMSE. For the subsequent DAPC analysis, two PCs and two discriminant functions were retained. The grouping assignment for individual strains by DAPC is listed in Table 1 (as I-SNPs). Within the five clusters (Fig 2(c)), C1 is a CBD dominant cluster that includes six strains (3, 4, 5, 6, 8, and 10-CBD), C2 includes three balanced strains (2, 7, and 9-balanced), and C3, C4, and C5 are THC dominant clusters that include two (13 and 22-THC), seven (1-balanced, 11, 15, 18, 20, 21, 23-THC), and five (12, 14, 16, 17, and 19-THC) strains, respectively.

**Fig 3:**
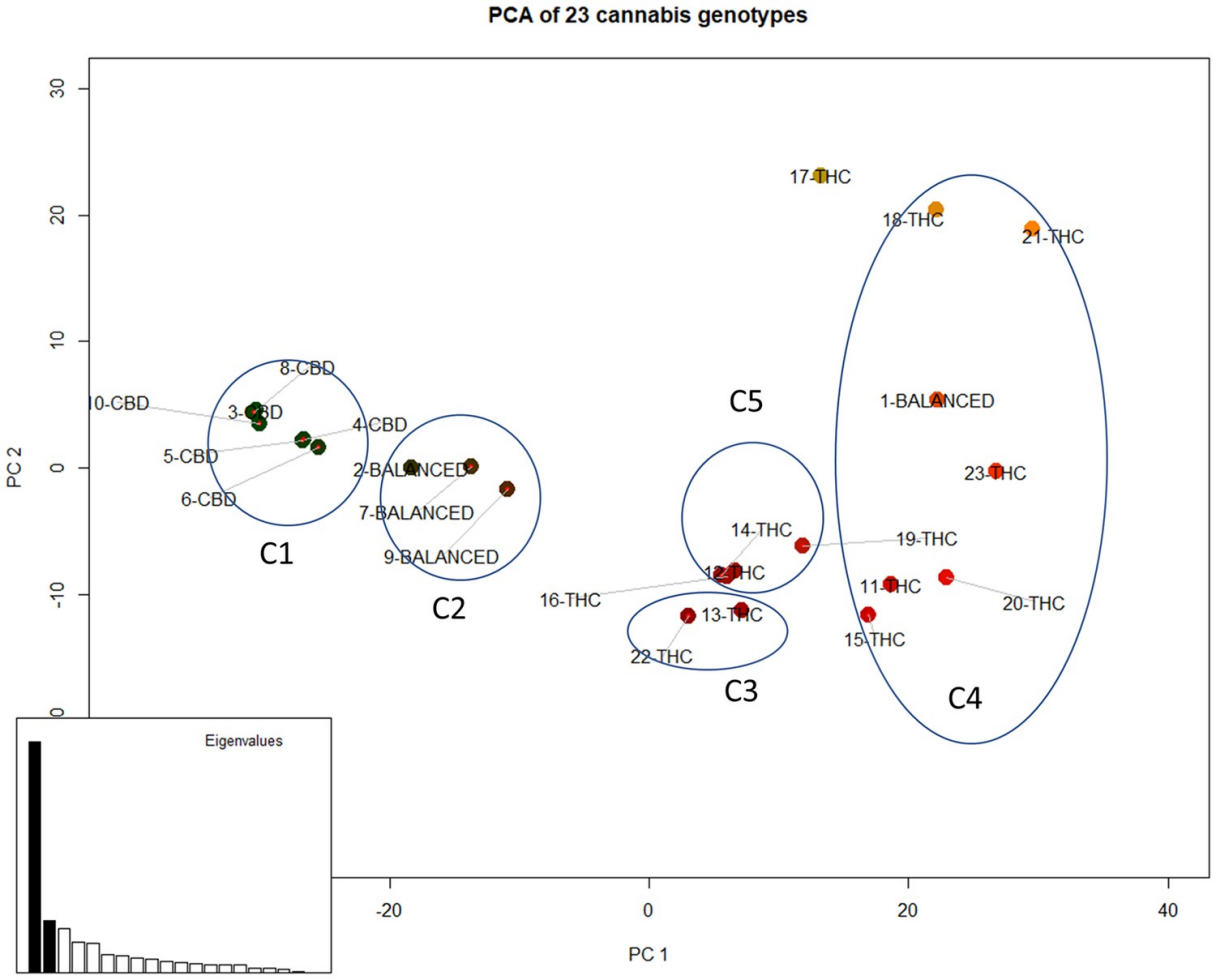
Scatter plot of 23 cannabis strains on PC1 & PC2 using 344 structural SNPs. Clusters indicated as C1, C2, C3, C4 and C5 correspond to I-SNPs in Table 1.

These multiallelic SNPs were also subjected to PCA, NJ tree, and hierarchical clustering analysis. In Fig 3, the 23 cannabis strains are plotted along PC1 and PC2, which account for 44.5% and 10.0% of the total variance, respectively. The proportions of explained variance are higher compared to the previous PCA results (18.4% and 11.5%) obtained using the whole set of SNPs. CBD dominant cluster C1 and balanced cluster C2 are on the left side of the scatter plot (PC1<0) and the THC dominant clusters C3, C4, and C5 are on the right side of the scatter plot (PC1>0). Notably, six CBD dominant strains are separated from three balanced strains, while they were previously combined in the analysis using the whole set of SNPs. In addition, two THC dominant strains 13-THC and 22-THC are separated from the CBD and balanced cluster, and instead placed closer to other THC dominant strains. Strain 1-balanced is closer to THC dominant strain regardless of whether the whole set of SNPs or 344 identified SNPs were used.

**Fig 4:**
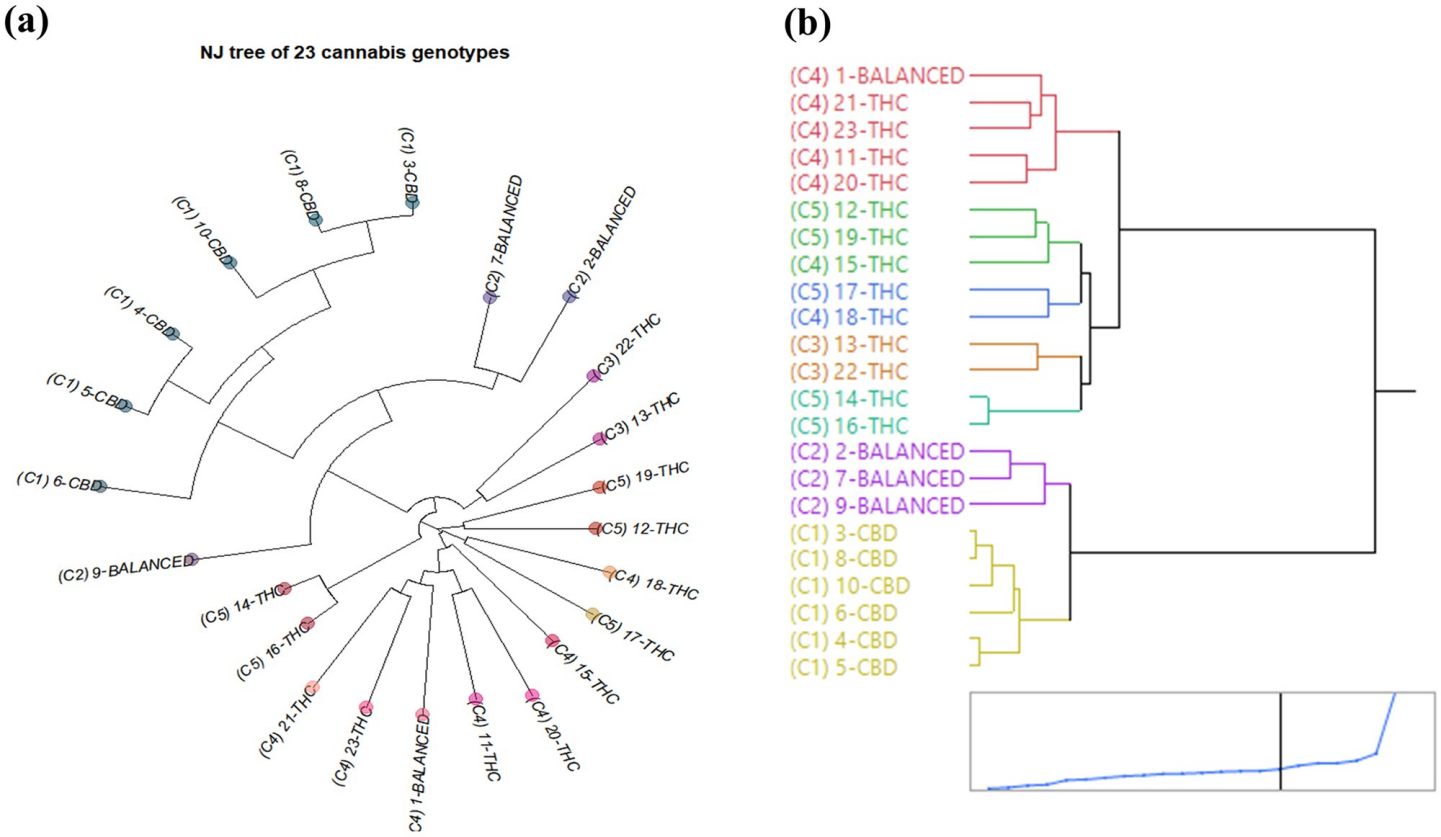
NJ-tree and hierarchical clustering using the 344 multiallelic SNPs (a) NJ-tree and (b) The dendrogram using hierarchical clustering by Ward’s method for 23 cannabis genotypes. Clusters indicated as C1, C2, C3, C4, and C5 corresponds to I-SNPs in Table 1.

The genetic structure from NJ-tree and hierarchical clustering using the 344 multiallelic are displayed in Fig 4, mostly congruent with that of DAPC. In the NJ-tree, all six CBD dominant strains are clustered together, with three balanced strains clustered closer on the same branch (Fig 4(a)). Most THC dominant strains are also clustered adjacent to strains within their own clusters. The dendrogram using hierarchical clustering by Ward’s method reveals two major groups, where one group is comprised of CBD dominant & balanced strains, and the other of THC dominant strains (Fig 4(b)). They are further separated into five subclusters, where CBD dominant and balanced clusters are consistent with the DAPC grouping results, and several THC dominant strains clustered differently. Two strains, 15-THC and 18-THC, were assigned to C4 using DAPC but are assigned closer to C5 in the dendrogram. Two other strains, 14-THC and 16-THC, were assigned to C5 in DAPC but are assigned closer to C3 in the dendrogram. The clustering results are congruent between DAPC and hierarchical clustering with an assignment agreement rate of 83% (19/23).

### Allele frequencies for 344 multiallelic SNPs in three chemotypes

**Fig 5:**
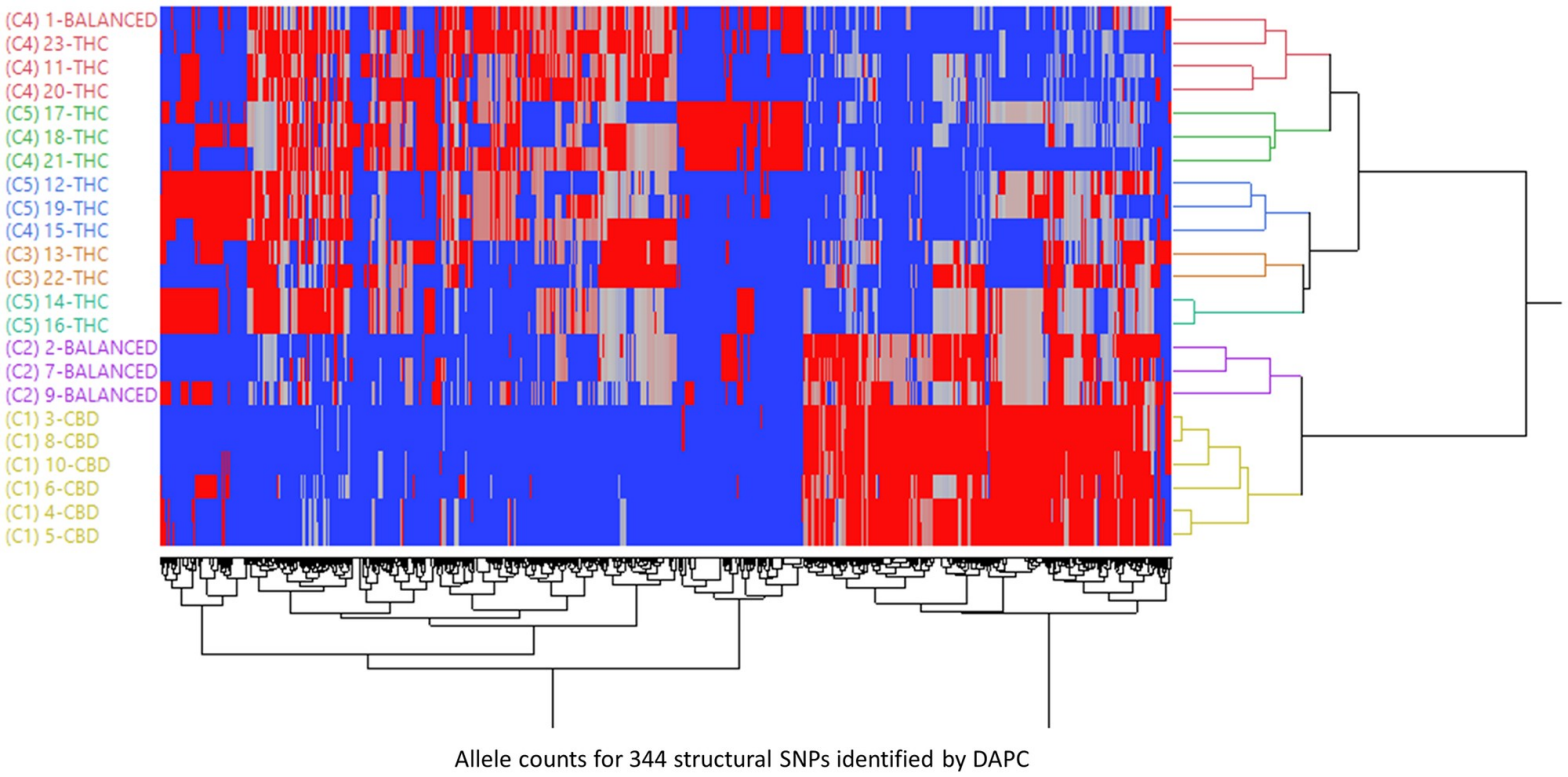
Hierarchical clustering of 23 strains based on the allele counts for 344 structural SNPs identified by DAPC.

DAPC identified 344 highly contributing SNPs (S1 Table). All the structural SNPs are multiallelic, among which 98.5% (339/344) are tri-allelic and the remainder 1.5% (5/344) are tetra-allelic. The dendrogram of 23 strains using hierarchical clustering based on the allele counts in the 344 structural SNPs (S2 Table) separated the strains into CBD dominant, balanced, and THC dominant strains, mostly corresponding to the grouping results of DAPC (Fig 5). The allele frequency was calculated by dividing the counts of that allele for all strains within the targeted group by the sum of the counts for each allele for that SNP within the targeted group. Allele frequencies of the structural SNPs were calculated for three major branches, each corresponding one of three chemotypes. (S1 Table). If 1-balanced strain was assigned to the THC dominant group as indicated by DAPC for allele frequency calculation, there are 87% (300/344) SNPs in CBD dominant clusters, 46% (157/344) SNPs in balanced clusters, and 11% (39/344) SNPs in THC dominant clusters that have one allele with allele frequencies > 80% (S1 Table). Among them, 140 SNPs shared same alleles with allele frequencies > 80% in CBD dominant strains (140/300) and balanced strains (140/157), which further indicated that CBD dominant strains and balanced strains closely share a gene pool. There are 38 SNPs that have one allele present in CBD dominant strains with allele frequencies > 80% and are not detected in THC dominant strains. There are 322 SNPs whose alleles that are present in THC dominant strains but were not detected in CBD dominant strains.

If the 1-balanced strain is assigned to the balanced group for allele frequency calculation, there are 87% (300/344) SNPs in CBD dominant clusters, 10% (36/344) SNPs in balanced clusters, and 13% (44/344) SNPs in THC dominant clusters that have one allele with allele frequencies > 80% (S2 Table). Among them, 32 SNPs shared same alleles with allele frequencies > 80% in CBD dominant strains (32/300) and balanced strains (32/36). There are 38 SNPs that have one allele present in CBD dominant strains with allele frequencies > 80% and are not detected in THC dominant strains. There are 321 SNPs whose alleles are present in THC dominant strains but were not detected in CBD dominant strains. Assigning the 1-balanced strain to the balanced group added more genetic diversity to the balanced group, and the effect of adding or deleting this strain for the THC dominant group in terms of allele frequency is small and can be neglected.

### BLAST analysis of 344 multiallelic SNPs

**Fig 6:**
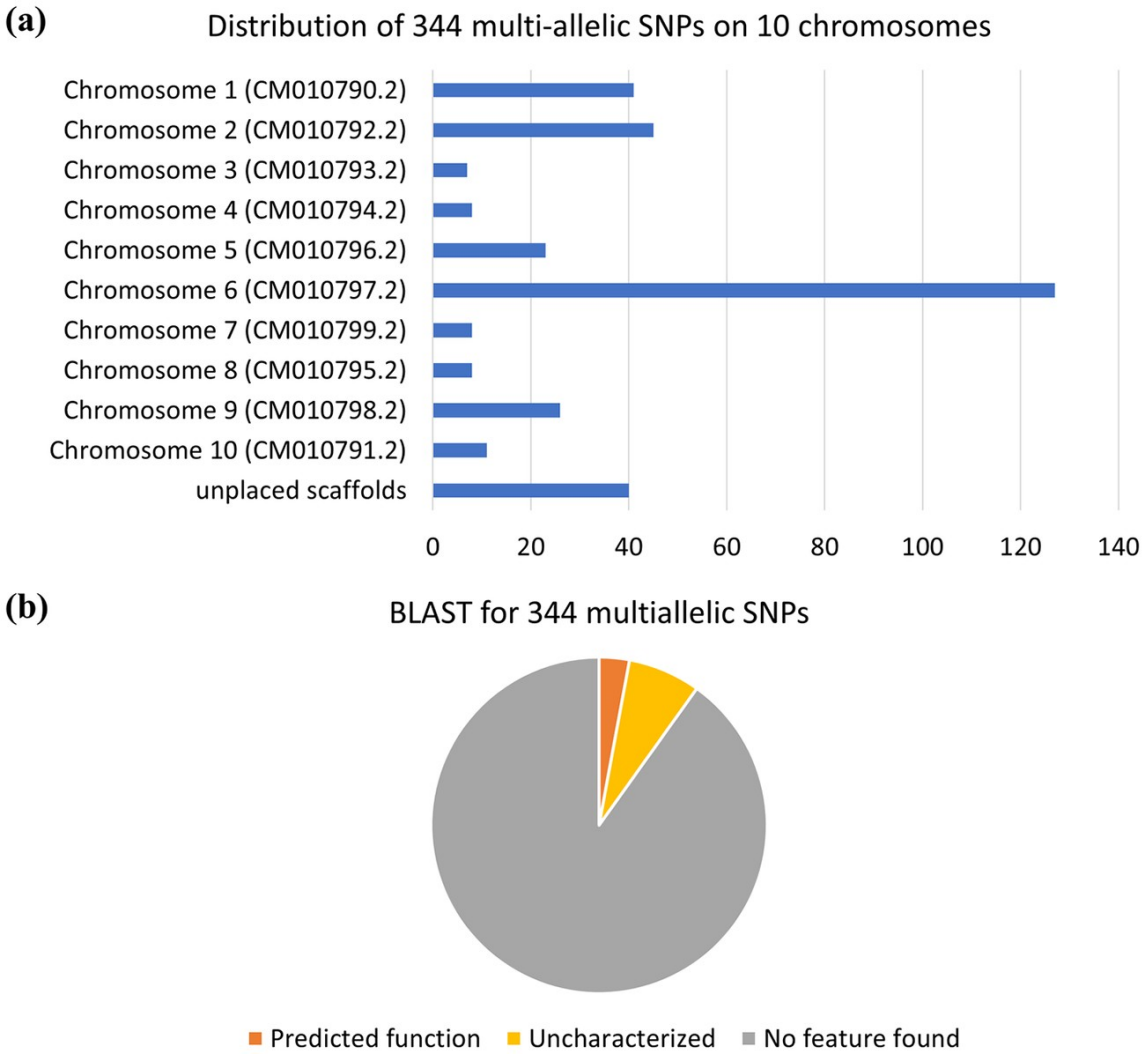
Features of 344 multiallelic SNPs **(a)** Distribution of structural SNPs on chromosome 1–10 and unplaced scaffolds. **(b)** BLAST results for structural SNPs against a fully annotated genome.

These 344 SNPs were spread across all 10 chromosomes (Fig 6(a)), indicating that commercially available cannabis strains in North America are significantly differentiated at a genome-wide level. The number of identified SNPs ranged from 7 to 127 on each genome, with 37% of the genetic variation occurring (127 SNPs) on chromosome 6, where CBDAS and THCAS are located [13]. The rest SNPs were spread over the remaining nine chromosomes. All ten chromosomes have genes related to the biochemical pathways of secondary metabolites, including cannabinoids, monoterpenes, and sesquiterpenes [13, 24, 47–51]. BLAST results showed that 90% (310/344) of these structural SNPs had no feature, 7% (24/344) are uncharacterized loci with unknown functions, and 3% (10/344) are predicted for certain functions (Fig 6(b)).

## Conclusions

Although the cannabis industry is rapidly advancing after the relaxation of legal restrictions in North America, the increasing number of THC dominant strains, CBD dominant strains, and balanced strains only adds confusion to the currently poorly understood genetic background of the thousands of varieties already in existence. Although there were only 23 strains included in this study, they covered the three typical chemotypes of cannabis strains currently available in the market. Leveraging as much genetic variation as possible using whole-genome sequencing, we identified 344 multiallelic SNPs that were used to investigate the genetic structure of 23 cannabis genotypes using DAPC, PCA, NJ tree, and hierarchical clustering, which provided consistent observations and groupings despite the differences in algorithms. The clustering results revealed that these 23 strains could be separated into five clusters, with one cluster containing six CBD dominant strains, another cluster containing three balanced strains, and the remaining three clusters containing 13 THC dominant strains and one balanced strain. CBD dominant strains and the balanced strains are closer genetically. This may be attributed to how medical interest in breeding for non-psychoactive, CBD-elevated strains (CBD dominant and balanced strains) has only recently been in vogue, resulting in an overlapping and less diverse gene pool for CBD dominant and balanced strains compared to the longer breeding history for THC strains. Some alleles are only present in CBD dominant strains or in THC dominant strains. More alleles present in balanced strains are shared with CBD dominant strains. One third of these structural SNPs are located on the chromosome containing THCAS and CBDAS. The remaining SNPs are located on the other nine chromosomes. An area of potential investigation is how the identified structural SNPs are associated with the production of other cannabinoids, mono- and sesquiterpenes, flavonoids, other compounds, or morphological characteristics.

Since the late 20th century, genetic methodologies have been developed for separating industrial hemp from drug-type cannabis for forensic purposes, thus differentiating CBD dominant and THC dominant strains [52–56]. For the past 20 years, with the extensive hybridization of THC dominant strains, many classification studies have focused on separating “Sativa” and “Indica” strains and many have suggested abolishing this vernacular [5–7]. The genotyping results of this study indicate that modern, extensively hybridized strains can still be separated using genome-wide information. As a powerful multivariate approach that investigates population structures based solely on genetic information, DAPC separated strains into clusters aligned with their chemotypes. Additionally, DAPC has the potential to sort the disordered genetic background of thousands of THC dominant strains by identifying the number of genetic clusters within THC dominant strains, describing clusters by interpreting group memberships, and identifying the contributing SNPs that have the potential to be used as genetic markers for strain classification and identification. This would require a concerted effort from the cannabis industry by contributing whole genome sequence data to public databases and by building a common taxonomy based on genomics. Optimally, the identified genetic markers can be used as genomic fingerprints in combination with chemical fingerprints and morphological characteristics for strain identification. These markers can be leveraged for strain selection in clinical trials and for manufacturing cannabis-based products and medicines.

## Supporting information

S1 Fig PCA of 23 strains using whole set of SNPs. (PDF)

S1 Table 344 multiallelic SNPs identified by DAPC. (XLSX)

S2 Table Allele counts for 344 structural SNPs identified by DAPC. (XLSX)
