# Supplementary material for: Classification of cannabis strains in the Canadian market with discriminant analysis of principal components using genome-wide single nucleotide polymorphisms
Source: PLoS One. 2021 Jun 28;16(6):e0253387. doi: 10.1371/journal.pone.0253387 (PMC8238227; doi:10.1371/journal.pone.0253387)
Supplement: S1 Fig — (PDF) [file pone.0253387.s001.pdf]

S1 Fig. PCA of 23 strains using whole set of SNPs

(a) P1&P2

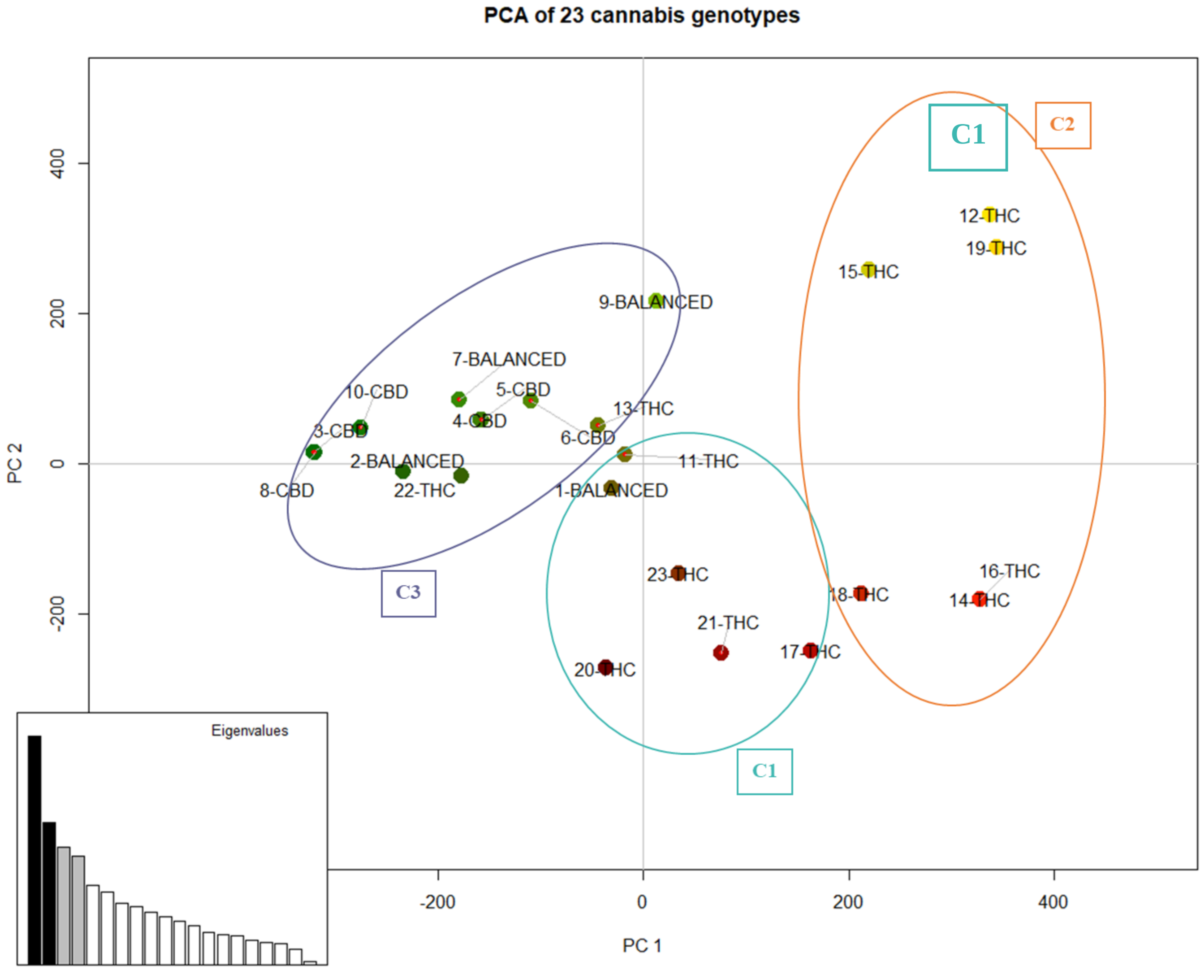

(b) P1&P3

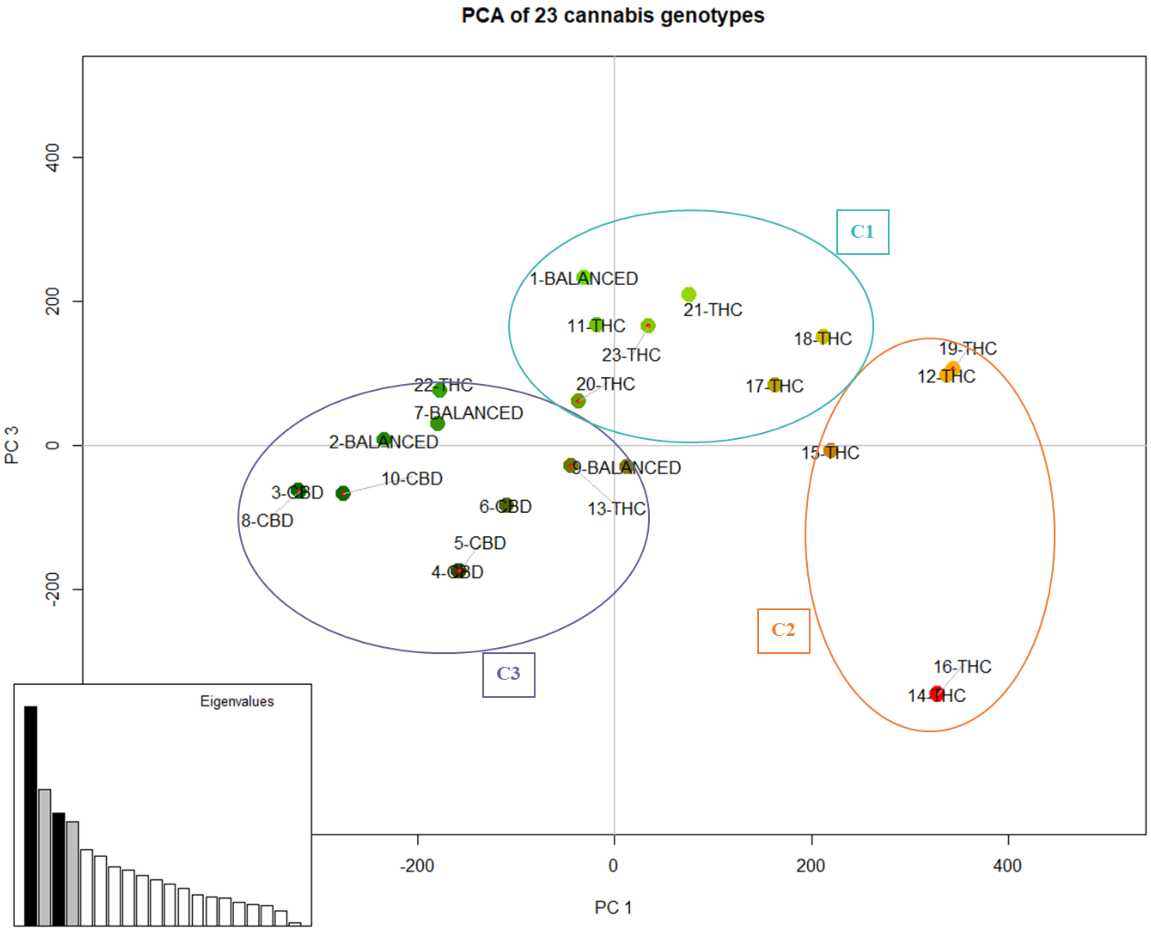
PCA of 23 cannabis genotypes
PC 3
PC 1
400
200
0
-200
-200
0
200
400
C1
C2
C3
1-BALANCED
21-THC
11-THC
23-THC
18-THC
19-THC
12-THC
20-THC
17-THC
22-THC
7-BALANCED
2-BALANCED
15-THC
9-BALANCED
10-CBD
3-CBD
8-CBD
6-CBD
13-THC
5-CBD
4-CBD
16-THC
14-THC
Eigenvalues

(c) P1&P4

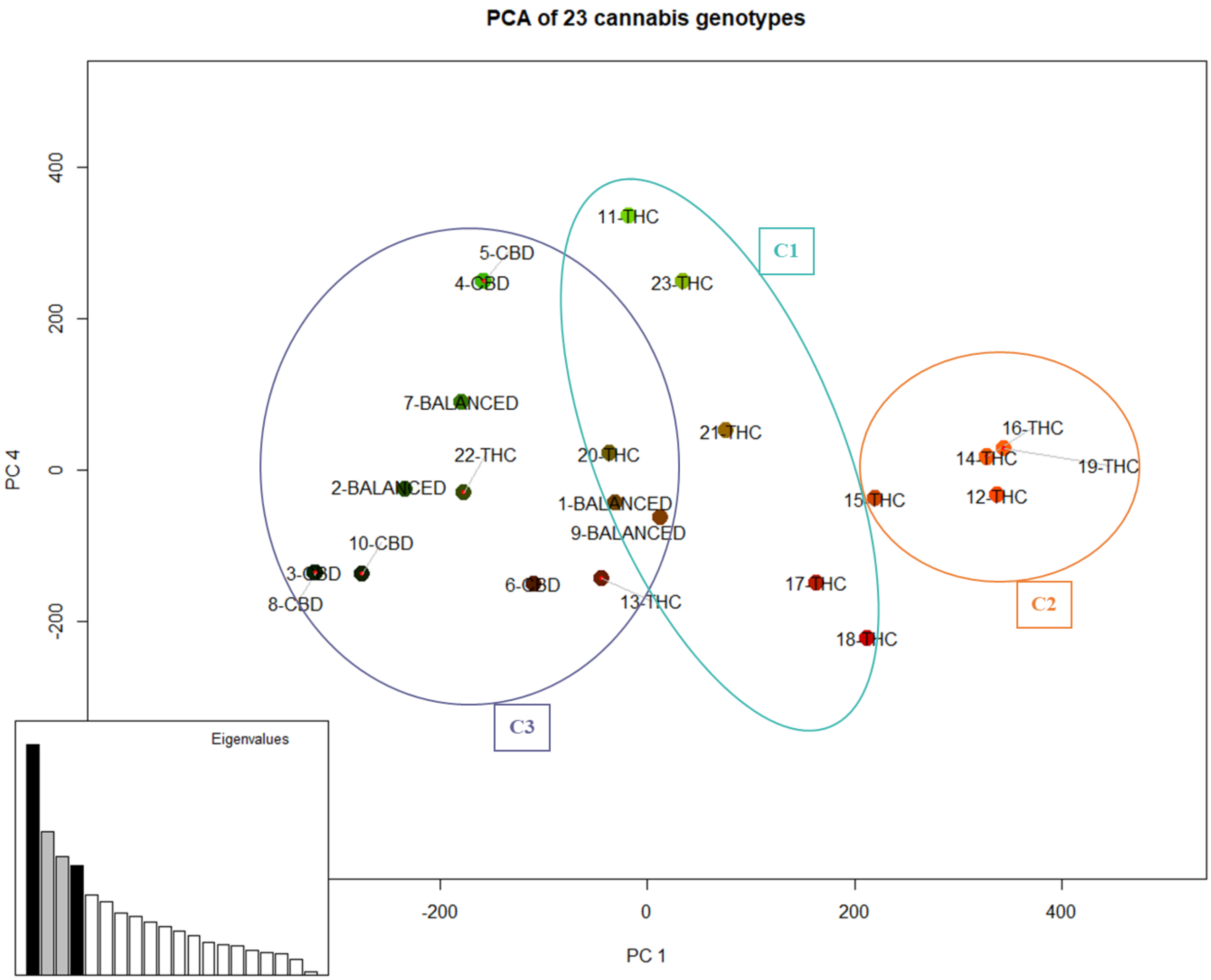
PCA of 23 cannabis genotypes
PC 4
PC 1
400
200
0
-200
-200
0
200
400
C1
C2
C3
11-THC
5-CBD
4-CBD
23-THC
7-BALANCED
21-THC
16-THC
14-THC
19-THC
22-THC
20-THC
2-BALANCED
1-BALANCED
15-THC
12-THC
9-BALANCED
10-CBD
3-CBD
8-CBD
6-CBD
13-THC
17-THC
18-THC
Eigenvalues

(d) P2&P3

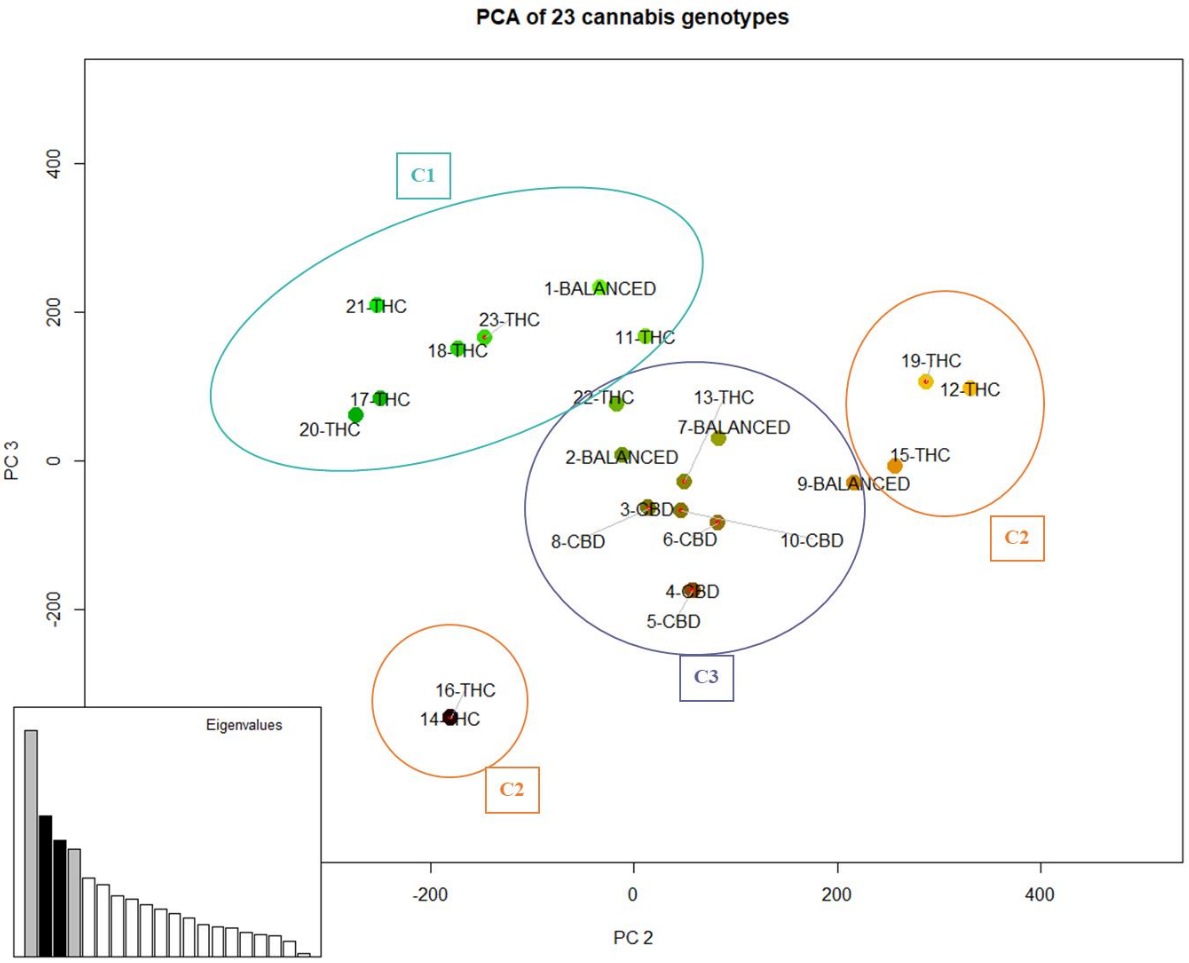
PCA of 23 cannabis genotypes
PC 3
PC 2
400
200
0
-200
-200
0
200
400
C1
C2
C3
C2
1-BALANCED
21-THC
23-THC
18-THC
11-THC
17-THC
20-THC
22-THC
13-THC
7-BALANCED
2-BALANCED
19-THC
12-THC
15-THC
9-BALANCED
3-CBD
8-CBD
6-CBD
10-CBD
4-CBD
5-CBD
16-THC
14-THC
Eigenvalues

(e) P2&P4

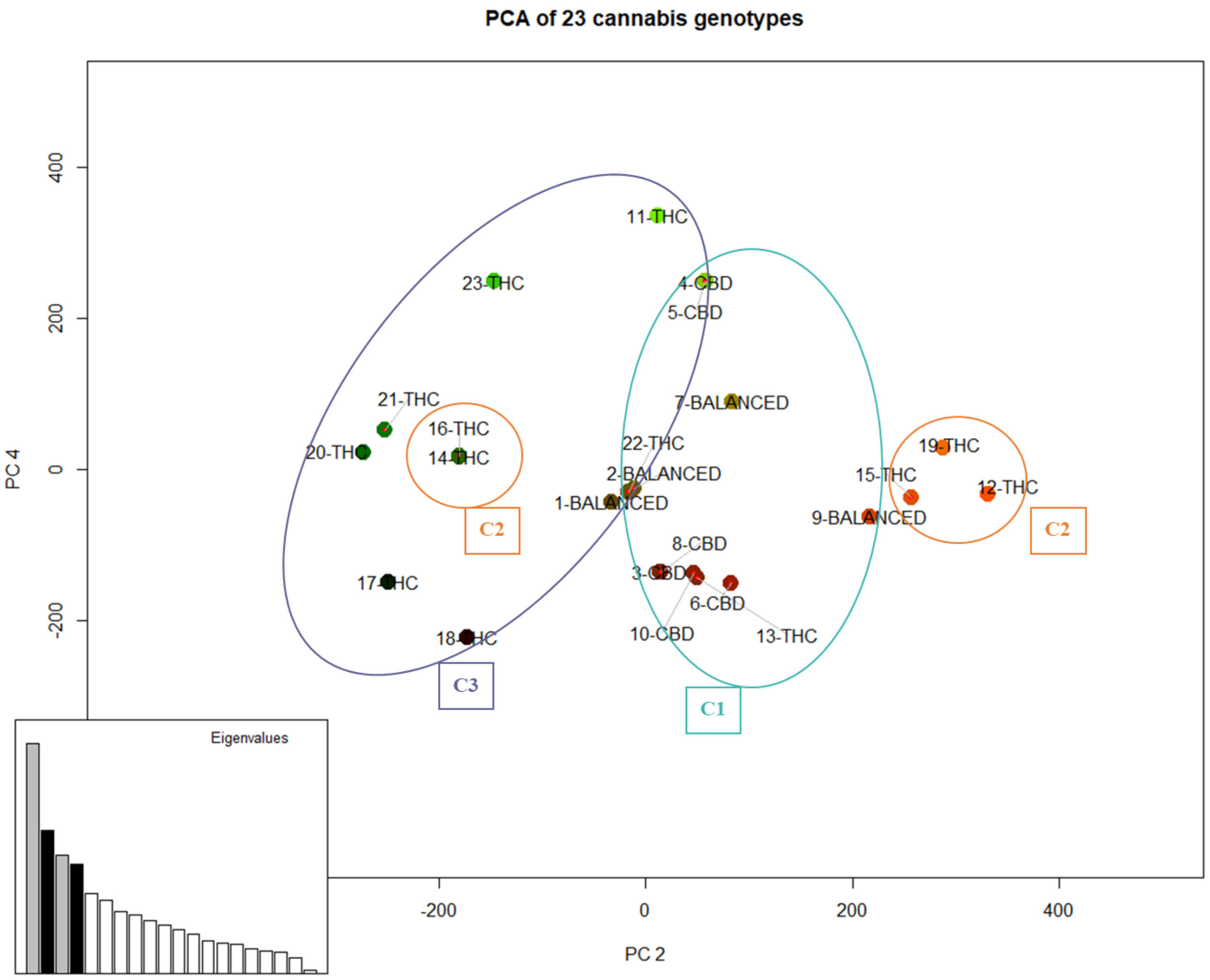
PCA of 23 cannabis genotypes
PC 4
PC 2
400
200
0
-200
-200
0
200
400
11-THC
23-THC
4-CBD
5-CBD
21-THC
16-THC
14-THC
20-THC
7-BALANCED
22-THC
2-BALANCED
1-BALANCED
19-THC
15-THC
12-THC
9-BALANCED
8-CBD
3-CBD
6-CBD
10-CBD
13-THC
17-THC
18-THC
C1
C2
C2
C3
Eigenvalues

(f) P3&P4

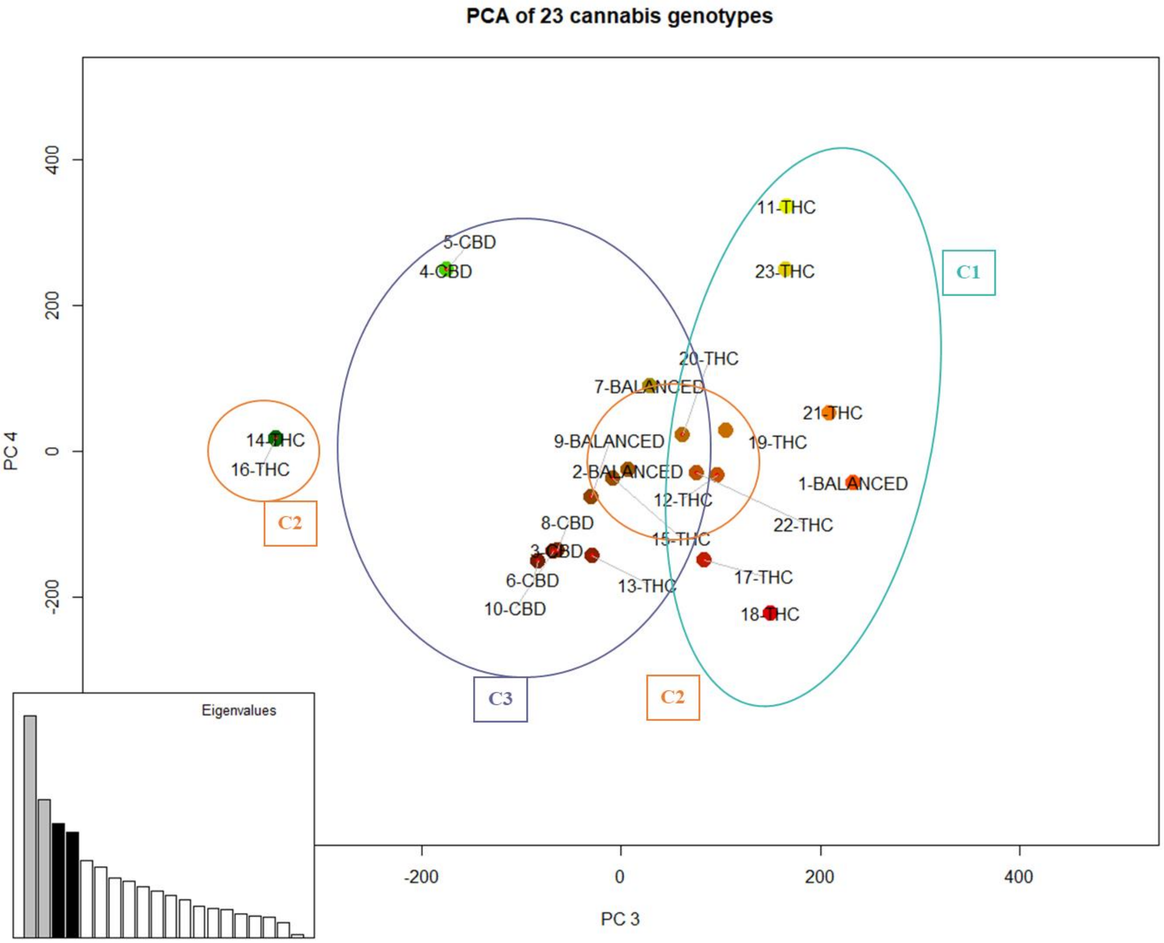

S1 Fig. Scatter plot of 23 cannabis strains on (a) PC1 & PC2, (b) PC1 & PC3, (c) PC1 & PC4 (d) PC2 & PC3 (e) PC2 & PC4 (f) PC3 & PC4 using 137,858 SNPs. Clusters indicated as C1, C2, and C3 correspond to W-SNPs in **Table 1**.
